# Cecal Volvulus Caused by a Strangulating Fallopian Tube: A Case Report and Brief Literature Review

**DOI:** 10.7759/cureus.114676

**Published:** 2026-08-17

**Authors:** Amanda YeQian Cui, Evan J Gorgas, Asha Shah

**Affiliations:** 1 Department of Surgery, Michigan State University College of Osteopathic Medicine, East Lansing, USA; 2 Department of General Surgery, Trinity Health Ann Arbor Hospital, Ypsilanti, USA; 3 Department of Trauma and Acute Care Surgery, Trinity Health Ann Arbor Hospital, Ypsilanti, USA

**Keywords:** bowel obstruction, cecal volvulus, fallopian tube, hysterectomy, laparotomy, salpingectomy

## Abstract

Cecal volvulus occurs when the cecum, ascending colon, and terminal ileum twist around the mesenteric pedicle. It is a rare cause of intestinal obstruction, and several associated factors include cecal hypermobility, previous abdominal surgery, adhesions, colonic tumors, pregnancy, abdominal masses, and congenital conditions such as Ehlers-Danlos syndrome. Without prompt recognition, cecal volvulus may progress to necrosis, gangrene, or perforation, resulting in sepsis and necessitating emergent operative intervention. Cecal volvulus secondary to strangulation by a fallopian tube is an even rarer entity that has been reported only twice in the literature. This case underscores the importance of considering gynecologic etiologies in the differential diagnosis of bowel obstruction, particularly in female patients.

## Introduction

Cecal volvulus is a rare but well-recognized cause of large bowel obstruction, accounting for only 1-2% of all bowel obstructions; however, it is the second most commonly encountered type of colonic volvulus after sigmoid volvulus [1]. The underlying anatomical predisposition results from incomplete fixation of the cecum and ascending colon to the posterior parietal peritoneum during embryologic midgut rotation, an anatomical variant found in up to 25% of the general population in autopsy studies [1]. Although this congenital predisposition is common, symptomatic volvulus is typically precipitated by additional factors such as advanced age, chronic constipation, pregnancy, adhesions, tumors, or prior abdominal surgery [2]. Outcomes are closely tied to timely diagnosis, as delayed recognition increases the risks of bowel ischemia, perforation, and mortality, particularly in older patients or those with comorbidities [3].

Among the many described precipitants of cecal volvulus, entrapment or strangulation by a fallopian tube is exceptionally rare. Cecal volvulus caused by a fallopian tube has been described only twice in the literature [4,5]. Notably, in both previously reported cases, the patients had a history of prior gynecologic surgery: one had undergone abdominal hysterectomy and the other unilateral oophorectomy. This raises the question of whether alterations in the length or mobility of the fallopian tube, or in its anatomical relationship to the right colon and mesentery following pelvic surgery, may predispose patients to this complication. However, given the limited number of cases, the precise mechanism by which a fallopian tube encircles the cecum or its mesentery remains poorly understood, as does the role that prior gynecologic surgery may play in this process.

Although very rare, this phenomenon represents another etiology of mechanical bowel obstruction in women and underscores the importance of considering gynecologic structures in the differential diagnosis of an acute abdomen in women with a history of pelvic surgery. It may also support the argument for complete extrauterine tubal removal during hysterectomy rather than partial removal, a practice already favored for its potential oncologic benefits, as complete salpingectomy may offer greater ovarian cancer risk reduction than partial salpingectomy [6]. We present a third case of cecal volvulus secondary to a strangulating fallopian tube and review the existing literature on this entity, including the proposed mechanisms, diagnostic considerations, and treatment approaches.

## Case presentation

A 61-year-old woman with a medical and surgical history of type 2 diabetes, gastroesophageal reflux disease, obstructive sleep apnea managed with continuous positive airway pressure (CPAP), anxiety, depression, laparoscopic cholecystectomy (2022), and laparoscopic radical supracervical hysterectomy (2009) for uterine fibroids presented to the emergency department with a one-day history of abdominal pain, nausea, and bloating. She continued to pass flatus after symptom onset and reported no changes in bowel movements.

**Figure 1:**
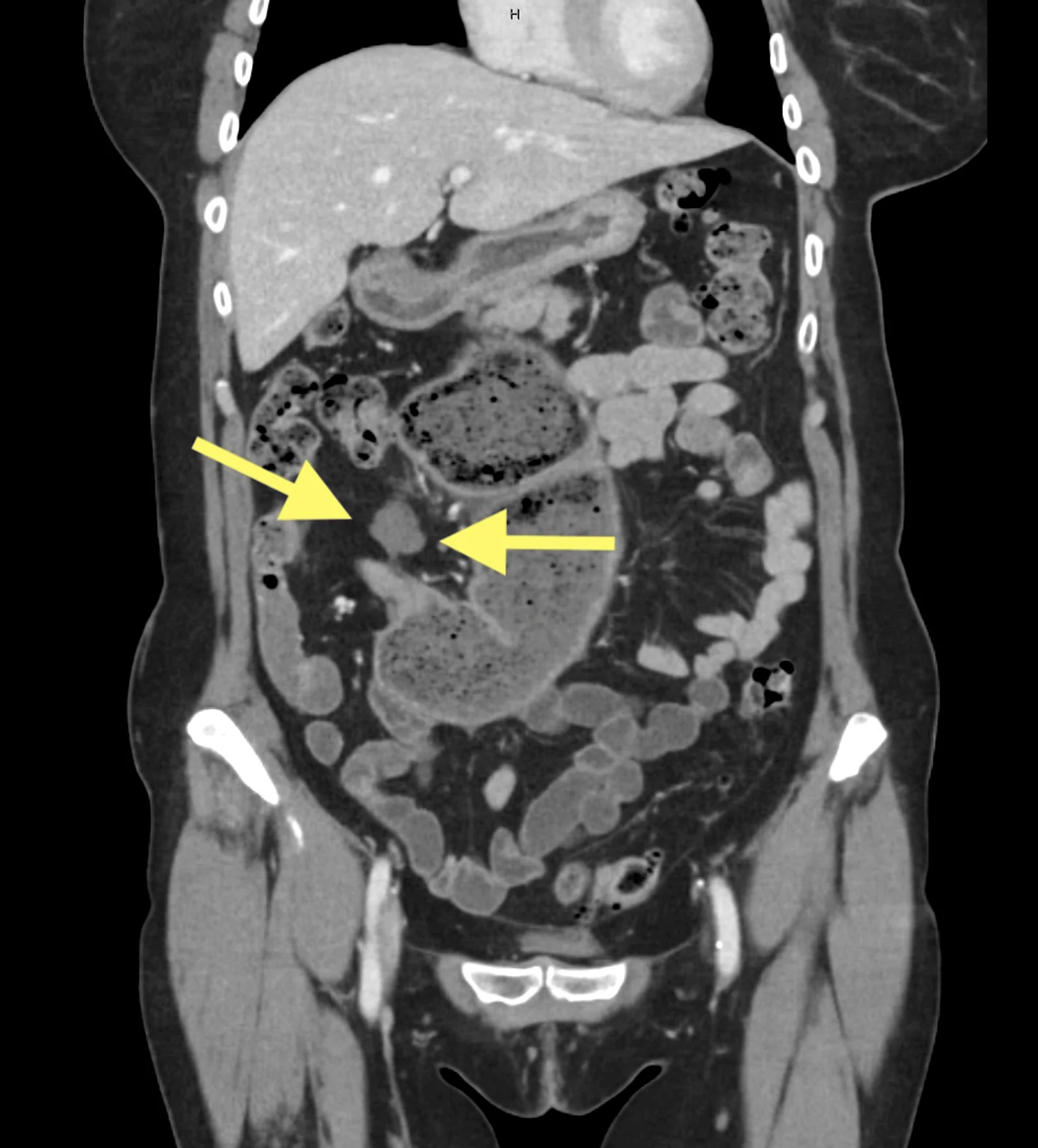
Coronal CT image showing marked dilation of the cecum and a transition point raising concern for cecal volvulus. Coronal CT image demonstrating marked dilation of the cecum extending superiorly and medially into the midabdomen. The yellow arrows indicate the site of torsion, with swirling of the mesenteric vessels (whirl sign), near the ileocecal transition zone, resulting in mechanical large bowel obstruction.

**Figure 2:**
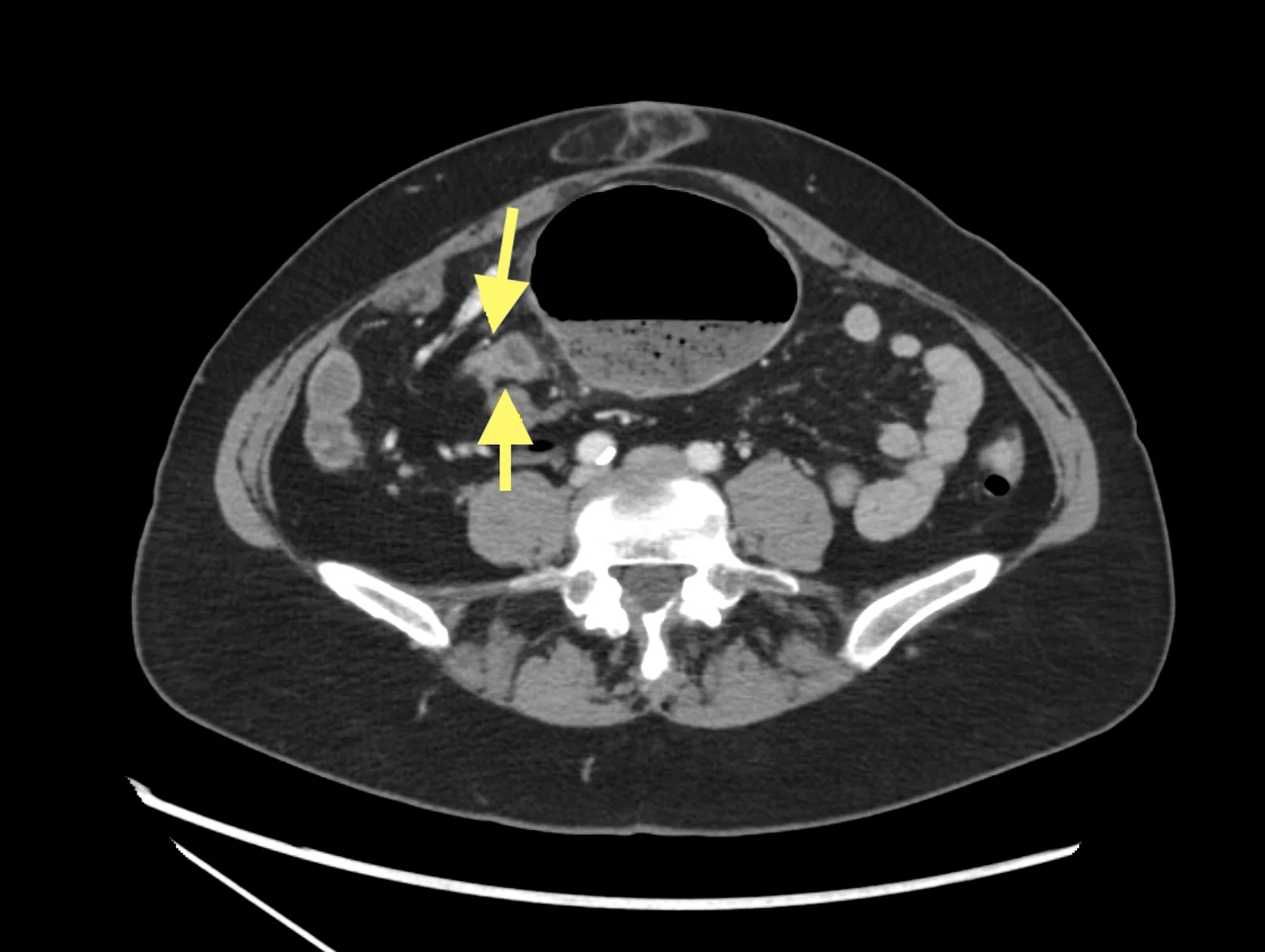
Axial CT image showing dilation of the cecum and a transition point raising concern for cecal volvulus. Axial contrast-enhanced CT image of the lower abdomen demonstrating a markedly distended, gas- and stool-filled cecum. The yellow arrows indicate mesenteric convergence at the transition point and adjacent fat stranding.

Her vital signs were notable for hypertension but were otherwise within normal limits. Laboratory studies showed a WBC count of 17.9 × 10³/µL, hemoglobin level of 15.7 g/dL, platelet count of 370 × 10³/µL, and lactate level of 2.0 mmol/L. Her abdomen was soft and mildly distended, with moderate tenderness in the right upper quadrant and mild tenderness in all other quadrants. A CT scan of the abdomen and pelvis with IV contrast (Figures 1-2) demonstrated dilation of the cecum to 12 cm, raising concern for cecal volvulus. No free fluid or free air was present. She was also incidentally found to have a small fat-containing umbilical hernia.

Because of the concern for cecal volvulus, the decision was made to proceed with urgent surgical exploration. During exploration, we encountered a distended cecum with early ischemic changes. As we explored further, we confirmed that the cecum was volvulized. Interestingly, the volvulus was caused by the midportion of the right fallopian tube wrapping around the mesentery. The fallopian tube appeared markedly thickened and hemorrhagic. Gynecology was consulted intraoperatively and agreed that salpingectomy was indicated. The fallopian tube was excised at the level of the ovary. Given the ischemic changes in the colon, distention of the cecum and ascending colon, and redundancy of the mesentery, the team proceeded with a right hemicolectomy with primary antiperistaltic ileocolonic anastomosis.

The patient’s postoperative course was unremarkable. A clear liquid diet was initiated on postoperative day (POD) 3, and she was discharged on POD 5. At her postoperative appointment one week after discharge, she demonstrated satisfactory recovery and did not require additional follow-up with the surgical team. According to notes from her primary care physician, she has experienced intermittent diarrhea since surgery. Pathologic examination of the right colon showed thinning of the colonic wall, compatible with volvulus. Examination of the right fallopian tube showed benign tubal tissue with infarction and hemorrhage, along with a small fragment of benign ovarian tissue.

## Discussion

Brief literature review

Following a further literature review, Table 1 summarizes cases of cecal volvulus secondary to a strangulating fallopian tube. The first case, reported in 2011, described an otherwise healthy patient with a remote surgical history of abdominal hysterectomy. The patient presented with an ileus after taking loperamide, ciprofloxacin, and dimenhydrinate for presumed gastroenteritis. Imaging findings were concerning for cecal volvulus, and she underwent emergency laparotomy. Intraoperatively, an ischemic cecum with volvulus at the level of the cecal mesentery was noted. The mesentery was tethered by the right fallopian tube. A right hemicolectomy with primary ileocolic anastomosis was performed, along with bilateral adnexectomy of the remnant fallopian tubes [4].

**Table 1:** Summary of available case reports of cecal volvulus secondary to fallopian tube strangulation, including patient demographics, diagnosis, and treatment modalities. GERD: Gastroesophageal reflux disease.

Author	Age (years)	Medical history	Surgical history	Diagnosis/operative findings	Treatment
Tee MC et al., (2011) [4]	62	None	Abdominal hysterectomy	Cecal volvulus at the level of the cecal mesentery, which was tethered by a remnant right fallopian tube	Right hemicolectomy with primary ileocolic anastomosis; bilateral adnexectomy of the remnant fallopian tubes
Hardy E et al., (2015) [5]	55	History of a benign left ovarian tumor	Left oophorectomy	Cecal volvulus caused by a strangulating right fallopian tube wrapped around the ascending colon	Right hemicolectomy; right salpingo-oophorectomy
Present case, 2026	61	Type 2 diabetes, GERD, obstructive sleep apnea, anxiety, and depression	Laparoscopic cholecystectomy (2022); laparoscopic supracervical hysterectomy (2009)	Cecal volvulus caused by the midportion of the right fallopian tube wrapping around the cecal mesentery, with early ischemic changes	

The second case, reported in 2015, briefly described a healthy patient with a history of a benign left ovarian tumor that had been resected. The patient presented with acute right lower quadrant pain. Imaging showed dilated loops of the small and large bowel concerning for obstruction. Colonoscopy revealed ischemic colitis at the level of the cecum, and the patient subsequently underwent emergency laparotomy. Intraoperatively, a cecal volvulus caused by a strangulating right fallopian tube wrapped around the ascending colon was found, with resulting ischemia and necrosis. The patient underwent right hemicolectomy and right salpingo-oophorectomy [5].

Among the three reported cases, the present patient was similar in age to the previously described patients (61 years compared with 62 and 55 years) and, like both prior patients, had a history of gynecologic surgery [4,5]. The prior cases involved a remote abdominal hysterectomy and a prior left oophorectomy, respectively, whereas the present patient had undergone a laparoscopic supracervical hysterectomy with presumed preservation of the adnexa [4,5]. All three patients presented with acute abdominal symptoms and imaging findings concerning for cecal volvulus or bowel obstruction. Intraoperatively, each case demonstrated a right fallopian tube encircling or tethering the bowel or its mesentery, with associated ischemic changes requiring right hemicolectomy. The present case therefore shares the characteristic operative findings and need for bowel resection described in the prior reports while further demonstrating strangulation of the cecal mesentery by a residual fallopian tube following supracervical hysterectomy [4,5]. The limited number of reported cases and the lack of detailed postoperative outcomes in the prior reports make broader comparisons of long-term outcomes difficult.

The mechanism by which the fallopian tube became wrapped around the cecal mesentery remains uncertain. Both previously reported cases occurred after gynecologic surgery, suggesting that altered pelvic anatomy or residual tubal mobility may contribute [4,5]. In the present case, preservation of the fallopian tube after supracervical hysterectomy may have left a mobile tubal remnant capable of reaching the adjacent cecal mesentery. Postoperative adhesions may also have contributed by altering the normal position or mobility of the adnexa; however, the operative findings in this case do not establish adhesions as the causative mechanism. Given the limited number of cases, it remains unclear whether residual tubal mobility, postoperative adhesions, altered pelvic anatomy, or a combination of these factors predisposes patients to strangulation of the bowel by a fallopian tube.

Mechanism of cecal volvulus

Cecal volvulus occurs in the setting of insufficient fixation of the cecum to the posterior peritoneum. During embryologic development, the midgut rotates 270° counterclockwise and subsequently becomes fixed to the posterior parietal peritoneum. Disruption of this process may result in inadequate fixation and a mobile cecum [7]. This anatomical variant is seen in up to 25% of the general population in autopsy studies; however, cecal volvulus accounts for only 1-2% of all bowel obstructions [1]. Although some individuals have this anatomical predisposition, the exact etiology is likely multifactorial and may involve advanced age, chronic constipation, previous abdominal surgery, tumors, pregnancy, adhesions, and congenital malformations [2].

Diagnosis

The preoperative diagnosis of cecal volvulus is difficult to establish clinically because patients present with variable and nonspecific symptoms similar to those of other abdominal conditions. Common complaints include abdominal pain, abdominal distension, vomiting, and absolute constipation, characterized by the inability to pass stool or flatus. Additional signs such as fever, tachycardia, hypotension, and generalized peritonitis may be present when there is concern for bowel compromise. Basic laboratory tests may help assess the severity of illness and the response to resuscitation; an increasing leukocyte count may raise concern for bowel ischemia [8]. It is important to note that the clinical presentation can vary, as our patient continued to pass flatus despite radiologic evidence of bowel obstruction.

Abdominal radiographs can identify bowel obstructions in approximately 70% of cases and classically demonstrate marked cecal dilation, often displaced from the right lower quadrant, with a large air-fluid level and an absence of gas in the distal colon [9]. However, CT can identify approximately 90% of cases of cecal volvulus and can often exclude other etiologies or assess the severity of bowel ischemia [10]. Characteristic CT findings include the “coffee bean,” “bird’s beak,” and “whirl” signs. The whirl sign consists of twisting mesenteric vessels and fat around the point of torsion, often with associated mesenteric edema [9]. On axial images, the coffee bean sign appears as a markedly dilated cecum containing air and fluid. These findings, particularly the whirl sign, are highly specific for volvulus. An abnormally positioned appendix in the central or upper abdomen is also commonly seen [10]. Preoperative identification of a strangulating structure causing the volvulus is challenging, and the structure is often recognized only intraoperatively.

Treatment

Surgical intervention remains the definitive treatment for cecal volvulus. Surgical techniques include manual detorsion, cecopexy, and right colectomy performed via laparotomy or minimally invasive approaches. Segmental resection is generally preferred, whereas nonresectional procedures may be considered in selected patients with viable bowel who are unfit for resection. Colonoscopic reduction has been reported; however, its success rate is low, and the risk of colonic perforation makes it inadvisable. Some authors have proposed laparoscopic cecopexy as a relatively safe procedure, although it is associated with a risk of recurrence [11]. In cases of ischemia or perforation, all nonviable bowel must be resected. Higher mortality rates have also been associated with coagulopathy, advanced age, peritonitis, and bowel gangrene [3].

Considerations

Minimally invasive surgery and laparotomy are both viable options, depending on the patient’s clinical status, comorbidities, age, operative findings, and the surgeon’s experience. Selected clinically stable patients may benefit from minimally invasive surgery because of reduced adhesion formation and a shorter hospital stay. However, laparotomy may be preferable in clinically unstable patients or when ischemia, perforation, marked bowel distension, dense adhesions, or difficult or unclear anatomy are encountered.

Concurrent bilateral salpingo-oophorectomy at the time of hysterectomy was historically common. However, because of growing concerns regarding hormonal deficiencies and other health consequences following premenopausal oophorectomy, there has been a shift toward ovarian preservation with opportunistic bilateral salpingectomy during hysterectomy. Population-based data suggest that tubal sterilization is associated with an approximately 28% reduction in ovarian cancer risk, whereas bilateral salpingectomy is associated with an approximately 65% reduction [6]. In 2015, the American College of Obstetricians and Gynecologists (ACOG) issued guidance supporting discussion of the potential benefits of salpingectomy during hysterectomy in women at population risk who are not undergoing oophorectomy. The present case raises the hypothesis that complete salpingectomy at the time of hysterectomy may reduce the potential for subsequent fallopian tube-related bowel obstruction; however, this cannot be established from the limited number of reported cases.

## Conclusions

This case demonstrates a rare occurrence of cecal volvulus caused by a strangulating fallopian tube. It underscores the importance of considering gynecologic etiologies as potential causes of bowel obstruction, particularly in female patients with a history of pelvic surgery. Although complete salpingectomy at the time of hysterectomy could theoretically reduce the risk of this complication by eliminating residual tubal tissue, this remains a hypothesis given the limited number of reported cases. Additional cases are needed to better understand the underlying mechanisms, optimal management, and potential strategies for preventing such obstructions.
